# Trading Health for Wealth: The Effect of COVID-19 Response Stringency

**DOI:** 10.3390/ijerph17238725

**Published:** 2020-11-24

**Authors:** Megan Cross, Shu-Kay Ng, Paul Scuffham

**Affiliations:** G40 Griffith Health Centre, Menzies Health Institute Queensland, Gold Coast Campus, Griffith University, Parklands Drive, Gold Coast 4111, Australia; m.cross@griffith.edu.au (M.C.); s.ng@griffith.edu.au (S.-K.N.)

**Keywords:** COVID-19, stringency index, GDP, infection rate

## Abstract

International governments’ COVID-19 responses must balance human and economic health. Beyond slowing viral transmission, strict lockdowns have severe economic consequences. This work investigated response stringency, quantified by the Oxford COVID-19 Government Response Tracker’s Stringency Index, and examined how restrictive interventions affected infection rates and gross domestic product (GDP) in China and OECD countries. Accounting for response timing, China imposed the most stringent restrictions, while Sweden and Japan were the least stringent. Expected GDP declines range from −8% (Japan) to −15.4% (UK). While greater restrictions generally slowed viral transmission, they failed to reach statistical significance and reduced GDP (*p* = 0.006). Timing was fundamental: governments who responded to the pandemic faster saw greater reductions in viral transmission (*p* = 0.013), but worse decreases in GDP (*p* = 0.044). Thus, response stringency has a greater effect on GDP than infection rates, which are instead affected by the timing of COVID-19 interventions. Attempts to mitigate economic impacts by delaying restrictions or decreasing stringency may buoy GDP in the short term but increase infection rates, the longer-term economic consequences of which are not yet fully understood. As highly restrictive interventions were successful in some but not all countries, decision-makers must consider whether their strategies are appropriate for the country on health and economic grounds.

## 1. Introduction

The significant impact of the COVID-19 pandemic highlights the need for swift, proactive responses to a rapidly evolving situation. Most mitigation strategies involve reduced human mobility (travel restrictions and mandatory quarantine), closures (of schools, businesses and public spaces), and changes to health policy (including testing regimes). The stringency of international government responses has varied substantially. While Sweden eschewed movement restrictions entirely [1], South Africa’s lockdown went further and included bans on the sale of alcohol and cigarettes [2]. Overall, most government interventions have slowed infection rates to varying degrees. However, closures and restrictions have ruinous effects on labor markets and the global economy [3,4], and the social and psychological consequences of lockdowns are profound [5,6,7]. As the cost of ‘flattening the curve’ pressures governments to relax restrictions, researchers are questioning which strategies have most effectively curbed viral transmission [8,9].

Comparisons of the overall efficacy of international responses have been difficult, given the heterogeneity of both the restrictions themselves and their specific political and social contexts. Fortunately, the need for a universal measure to compare the strictness of international responses was met by the Oxford COVID-19 Government Response Tracker (OxCGRT) [10], which assimilates data on 18 indicators (including closures and policy changes) to track governments’ pandemic responses. The OxCGRT’s Stringency Index (SI) is a composite score from 0 to 100 that considers the strictness of containment strategies (closures, movement restrictions) and information campaigns [10]. It is particularly useful because it allows direct comparisons of countries’ responses to the pandemic. When examined with epidemiological and economic data, it enables an investigation of the relationships (or lack thereof) between response stringency and both economic and infection outcomes. This allows us to consider whether the most stringent government restrictions have been the most effective responses to COVID-19.

Given the potentially devastating economic consequences of strict lockdowns, this work investigates the effect of intervention stringency on infection rates and gross domestic product (GDP, which is the best measure of the total output produced within a jurisdiction). By examining the stringency indices, case rates and predicted GDP changes in countries with both similar and different socioeconomic situations, we aim to verify whether, as predicted, more stringent responses are more effective than lenient ones in reducing viral transmission. Similarly, we investigate whether stricter responses do indeed inflict greater economic damage. As governments work to balance economic and human health amid the feared, and actual, ‘second wave’ of infections, this analysis provides information for decision-making on future stringency [11].

## 2. Materials and Methods

### 2.1. Data Sources

Stringency index data covering 1 January to 6 July 2020 were downloaded from the OxCGRT [10]. The complete COVID-19 dataset from the Oxford Martin School is updated daily and was downloaded on 23 July 2020 [12]. Countries’ predicted annual GDP growth was obtained from the Organisation for Economic Co-operation and Development’s (OECD’s) Real GDP double-hit scenario forecast [13], which considers all goods and services produced in a year and accounts for both the global economic climate and that of the individual country.

#### 2.1.1. Analysis Timeframes

On 1 January 2020, there were 27 confirmed COVID-19 cases; by 1 June, there were 6.22 million [12]. Seven weeks later, on 23 July, this had more than doubled to 15.21 million [12]. Thus, to account for the geographic spread of COVID-19, we considered data spanning both the first seven months of the pandemic (1 January to 23 July 2020) and a shorter timeframe, arguably at the height of the pandemic (1 June to 23 July 2020). This narrower timeframe was selected to both account for the geographic spread of COVID-19 and to avoid biasing the analysis away from those countries whose stringency indices remained low (or at zero) because the virus had not yet reached them in the earlier months.

#### 2.1.2. Countries of Interest

For simplicity, we first considered the stringency indices of the OECD countries and China, then focused on at least one country from each major geographic region. Since the vast differences in healthcare, political systems and socioeconomics complicate comparisons, we included three Nordic countries in this shortlist. Although they are distinct nations, Sweden, Finland, and Norway are culturally, politically and economically more similar than other nations with a shared geography and benefit from comparatively high-quality healthcare [14,15]. Thus, their inclusion supports a stronger interpretation of the effects of different stringency responses.

### 2.2. Statistical Analysis

Statistical analyses were performed in Stata/SE16.0 (StataCorp, College Station, TX, USA). We considered the following measures to describe the characteristics of response stringency: (a) maximum stringency index score (SI_max_); (b) time to respond to the pandemic (days from 1 January to SI > 0); (c) time to respond to first local case (days); (d) time to (first) maximum score (days since 1 January to reach the (first) SI_max_ if there is more than one mode); (e) response escalation (days since SI > 0 to reach the (first) SI_max_); (f) speed of escalation (SI_max_ divided by response escalation); (g) number of days at maximum score; (h) area under curve of SI time graph (AUC; quantifies stringency strength and duration); (i) average stringency strength (AUC_av_; equals AUC divided by the response duration).

Different multilevel models were used for analyzing data of different variable types. Multilevel mixed-effects negative binomial regression models were used on country-level data to explore the association between response stringency and infection rates, with the assumption of random continental effect and each country’s population treated as an exposure factor. To explore the association between response stringency and the impact of COVID-19 on GDP, we adopted multilevel mixed-effects linear regression models, assuming random continental effect, to analyze changes in annual GDP for 2019–2020 forecast under a double-hit scenario of COVID-19 [13]. Likelihood-ratio tests were used to assess the significance of continental heterogeneity in infection rates or GDP impact and to compare multilevel negative binomial versus multilevel Poisson models for modelling the number of infections.

## 3. Results

### 3.1. Stringency Indices of China and OECD Countries

Detailed results are available in Table A1 in Appendix A. Maximum stringency indices (SI_max_) for the 37 OECD countries plus China, 1 January to 6 July 2020, range from 46 to 96 out of 100, with a mean of 81 (Table 1 and Table A1). On average, countries took 31 days from 1 January to respond to the developing pandemic and many implemented precautionary restrictions before local cases were confirmed. Nevertheless, response initiation and escalation varied, as did the time each country spent under its strictest restrictions. For example, while Canada spent only three days at SI_max_, the USA was there for 86 days. Further, Table A1 shows a significant continental effect for time to maximum score (ICC = 0.742; 95% CI: 0.38–1.10), which indicates that the between-continent variability accounts for 74.2% of the overall variation here. Continents also differ in both time to maximum stringency (*p* < 0.001) and response escalation (*p* < 0.001).

The AUC and AUC_av_ (Table 1 and Table A1) consider average stringency over January–July of the pandemic and each country’s response, respectively. Higher AUC values indicate restrictions that were both more stringent and active for longer periods. Both timeframes are useful, given the continental effect observed, the staggered detection of first cases in various regions, and the unique situation in each country.

#### 3.1.1. Highest Stringency Responses

Given that the virus was first detected in China, it is unsurprising that the Chinese Government’s response was both the fastest and the longest (Table 1 and Table A1). While its stringency was moderate (SI_max_ = 82), China’s interventions escalated relatively rapidly (86 days to SI_max_; Table A1) and maximum levels were maintained for a quarter of the total response period. Thus, China had the greatest overall stringency when duration was considered (AUC = 12,391) and the second-highest average daily stringency (AUC_av_ = 66; Table A1). New Zealand imposed the most stringent restrictions (SI_max_ = 96), but took longer to reach SI_max_ and spent a shorter period there. Thus, its overall stringency is moderate (AUC = 7466).

In South America, Chile and Colombia are notable. Both countries had highly stringent responses (SI_max_ 89 and 91, respectively), but while Chile waited 74 days to respond, Colombia acted faster (21 days). Both took longer than average to reach SI_max_ and spent fewer days there, but diverged in response duration: Colombia’s strict response spanned an extended period, and thus it is the second-most stringent overall (AUC = 10,168).

#### 3.1.2. Lowest Stringency Responses

Sweden’s response to the pandemic received much scrutiny [1]. The country has the lowest maximum stringency (SI_max_ = 46) and one of the shortest responses. Although Sweden waited 37 days to act, 70 days of its 124-day response were spent at SI_max_. While it has the lowest overall stringency (AUC = 4861), its average daily stringency is not the lowest—nor is it in the bottom three (Sweden AUC_av_ = 39; lowest value is Japan: AUC_av_ = 31). (See the following Table 1).

### 3.2. Stringency Indices and Infection Rates

**Figure 1:**
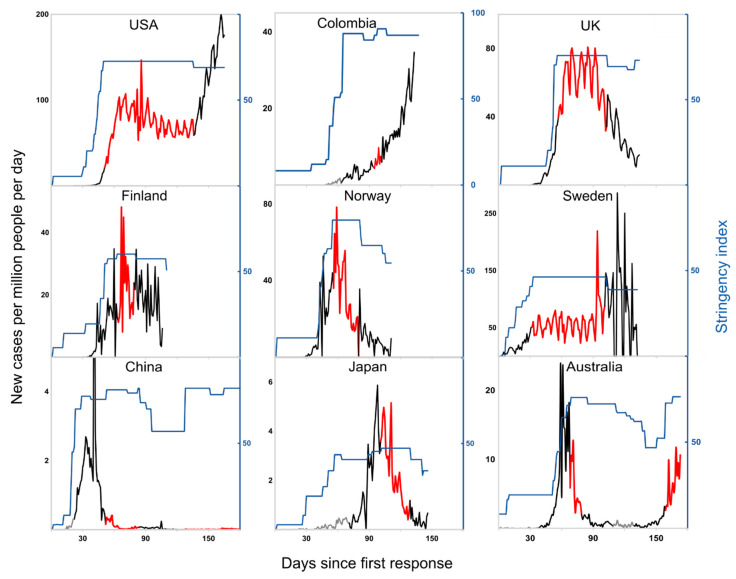
Stringency index and infection rates. The rate of new cases is shown in black for periods of one month before and one month after a period at maximum stringency index (SI) [10,12], which is highlighted in red. SI over the same period is overlaid in blue and scaled from 0 to 100 in all plots to enable comparisons.

At least one country from each geographic region was selected for further analysis. These included the USA and Colombia from the Americas, China and Japan from Asia, Australia from Oceania, and the UK, Sweden, Finland and Norway from Europe. Figure 1 and Table 2 examine the rates of new cases for one-month periods surrounding each country’s time at SI_max_. Overall, case numbers in most countries decrease during or shortly after maximum stringency, given a lag in lockdown efficacy. Exceptions include Sweden, Colombia, Australia, where a double-hit scenario is observed, and the USA. Although infections in the USA decreased slightly under maximum restrictions (Figure 1), its relaxation from SI 73 to 69 coincides with a rapid increase in cases. The country reported a daily average of 2.6 cases per million before restrictions escalated, 72.9 cases per million at SI_max_, and ~130 per million by the end of July (Table 2).

### 3.3. Risk Factors and Cumulative Infections

We examined the risk factors potentially associated with the number of cumulative infected cases up to 23 July 2020, using the full sample of 38 countries. To account for the progression of the pandemic, the statistical analysis considered a shorter period: 1 June to 23 July (Table 3). Here, the infection rate is higher when governments’ times to respond to the pandemic (RR = 1.028; 95% CI: 1.006–1.050; *p* = 0.013) and reach SI_max_ (RR = 1.072; 95% CI: 1.023–1.124; *p* = 0.004) are longer. Thus, those who respond faster and implement stringent interventions sooner appear most likely to reduce infection rates.

### 3.4. Stringency Indices and GDP Forecast

**Figure 2:**
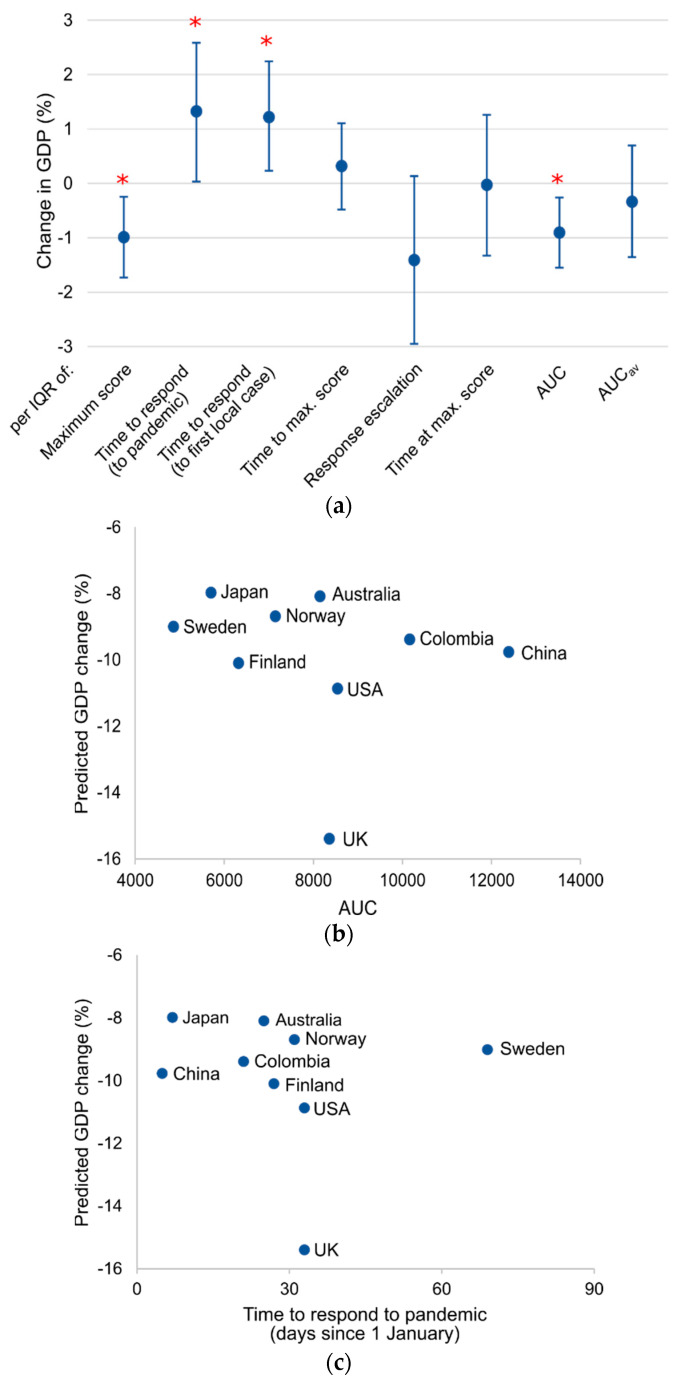
The effect of COVID-19 responses on annual GDP growth rates. Annual GDP growth rates were estimated by the OECD.13 (**a**) Results of multilevel mixed-effect linear regression models of change in annual growth rate 2019–2020 per interquartile range (IQR) due to a double-hit scenario (38 countries; multivariate analyses adjusted for GDP). Error bars show 95% confidence intervals; * *p* < 0.05. The difference between the 2019 and 2020 growth rates is shown against (**b**) response stringency and duration (area under the curve; AUC) and (**c**) the time for each country to respond to the pandemic (stringency index > 0); this includes both preventative restrictions that may have preceded confirmed cases in those countries.

Countries’ annual GDP growth rates for 2019–2020 were estimated by the OECD’s Real GDP forecast [13], which reports annual growth compared to the previous year, allowing comparisons as a relative percentage. Multilevel mixed-effect linear regression models examining the change in predicated growth for the 38 countries (Figure 2a; Table A2) found that higher SI_max_ (*p* = 0.012) and AUC (*p* = 0.006) are related to greater reductions in GDP. Similarly, faster responders are linked to decreasing growth (*p* = 0.044).

When response duration is considered, China was the most stringent overall and its economy is expected to decrease by an average of 9.8% (Figure 2b). Similarly, Colombia responded early, and its highly stringent restrictions linked to a 9.4% decrease. The greatest change in GDP is the 15.4% decrease predicted for the UK, which initiated a response two days after its first confirmed case (Table 1). While the USA escalated restrictions to approximately equal stringency to the UK (Figure 2b), its predicted GDP decrease is 10.9%. The annual growth rates of Japan (–8%) and Australia (–8.1%) are expected to change the least. Notably, both countries responded rapidly to the pandemic (Figure 2c). The three Nordic countries have predicted GDP declines of 8.7–10.1%. Finland responded to the pandemic first (Figure 2c) and its GDP is predicted to decrease the most. While the slowest responder, Sweden, introduced low-stringency interventions, Norway’s were the most stringent and its GDP is predicted to change the least (–8.7%).

## 4. Discussion

International responses to COVID-19 have been diverse. While strategies that suppress human movement and close public spaces generally decrease infection rates, their economic effects are immediate and severe. Thus, this work examined whether more stringent restrictions are linked to lower infection rates and what effect stringency has on GDP.

### 4.1. Rapid Responses Are Related to Decreased Infection Rates

A geographic effect was observed in how rapidly countries escalated their responses (Table 1 and Table A1). Generally, European countries increased their stringency faster, likely because the pandemic’s geographic origins allowed some nations more time to consider their strategies. Indeed, many countries implemented interventions before local cases were confirmed (Table A1). Infection rates were higher where governments were slower to initiate and escalate restrictions; however, no significant relationship exists between stringency and case rate. Instead, infection rates are related to response timing and escalation. 

### 4.2. Rapid Responses Affect GDP

The importance of timing is also highlighted by the GDP data, which link faster and more stringent responses to lower annual growth rates. This is likely to be a direct result of business closures and workforce immobilization. In the short term, the imposition of strict restrictions halts economic productivity, but where governments allow businesses to remain open, the economy is allowed to function ‘normally’ for a longer period (assuming that there is consumer demand). The GDP data were published on 10 June 2020 and thus, the impact on GDP does not account for the knock-on long-term economic effects of delayed responses: waiting too long to impose restrictions increases infection rates, which affects the workforce and healthcare system and could impact the economy if critical workers (or a significant proportion of the workforce) succumb to the virus [16,17].

Beyond the loss of human capability to COVID-19 mortality [18], higher infection rates could prolong the increased pressure already placed on healthcare systems. This has economic implications, particularly for countries with state-subsidized medical services (e.g., Medicare in Australia), which will be required to divert increased funding to bolster healthcare. Further, the emergence of ‘long COVID’ [19] suggests that a subset of the global population will require longer-term care for chronic side-effects of the virus, which will further impact states’ medical funding models. In addition, 20% of long COVID sufferers report lower quality of life and significant ongoing health problems [19], which could affect their participation in the workforce and the state’s economic productivity. Thus, while delayed responses may support economic stability in the short term, the detriment to human health may have a knock-on effect in the long term that ultimately affects the financial position of the state.

Further, the economic success of countries that rely on exports is arguably vulnerable regardless of their internal COVID-19 status. These states depend on international borders remaining open to shipments and on there being staff to receive goods and process payments. If a country does not impose restrictions, thus allowing production to continue and the local economy to continue unchanged, it may still face economic difficulties in the longer-term if goods for export cannot be delivered because client states’ borders are shut to mitigate infection [20]. This highlights the interconnectedness of the global economy and the potential longer-term consequences of delayed lockdowns for global trade.

This analysis also does not consider governments’ economic stimuli, which vary between jurisdictions [21]. Thus, given the complexity of the international economy, it is challenging to consider the relationship between GDP and stringency in a short-term vacuum.

Rapid, highly stringent responses decrease infection rates, thus easing the healthcare burden and allowing the resumption of ‘normal’ activity sooner. However, this approach depends on rapid population compliance and the capabilities of the supporting infrastructure. It is affected by each nation’s individual circumstances—its population and the nature of its labor market, social structure and health care system. Thus, to account for these differences, we considered Nordic countries, where national commonalities support a more even assessment [14].

### 4.3. Nordic COVID-19 Responses

Despite the attention it received for an apparently laissez faire response, Sweden’s strategy was carefully considered and of a similar average daily stringency to Finland’s (AUC_av_ of 39 and 38, respectively) [22]. Finland responded the fastest and had the lowest case rate under SI_max_; Sweden’s average rate was triple that and increased over this period (Figure 1). Thus, as expected by critics [23], the country’s low-stringency intervention was ineffective in slowing transmission and the number of cumulative infections in Sweden remains higher overall [12].

The Swedish approach was touted to avoid the economic fallout of lockdowns [23,24]. However, its economy is predicted to suffer more than that of Norway (Figure 2), which implemented the most stringent restrictions of the three countries. As Sweden’s cumulative mortality was almost 10 times those of its neighbors (561 per million vs. 59 and 47 per million in Norway and Finland, respectively, 23 July 2020) [12], the ultimate success of this strategy remains questionable, given the potential longer-term effects on the workforce. Further, given the highly interconnected nature of the global economy, national GDP may inevitably be affected by fluctuations in other countries and thus, the benefits of prioritizing local economic damage control may not balance the cost to human life.

### 4.4. Trading Human vs. Economic Health

The weighting of human versus economic health is complex. The emerging consensus is that the long-term effects of coronavirus mortality will outweigh the shorter-term economic impacts of lockdowns [25,26,27]. However, these conclusions are challenged by the inherent difficulty of modelling unknown outcomes: rather than mortality, the modelling of deaths averted is required to support solid conclusions. Without this baseline, validation of current progress remains uncertain. Further, deaths and their impacts will vary under a range of hypothetical scenarios with competing risks; thus, it is difficult to conclude whether governments have made the ‘right’ decisions. Learnings from previous pandemics reveal one fundamental factor: human behavior [28]. Indeed, there is little point keeping businesses open if customers stay home—and restrictions are only effective at curbing transmission if citizens comply.

### 4.5. Stringency in Context

Sweden’s response relies on social responsibility and the willingness of citizens to follow government recommendations [22,29]. Opposition to highly stringent lockdowns may emerge in response to the economic and sociological consequences and it is notable that we initially linked higher democracy indices to increased infection rates (Table 3). Given recent debates on COVID-19 restrictions and civil liberties [30], this prompts discussion of the public’s role in the success of pandemic interventions. SI is a composite measure that does not account for nuances in sub-national government responses and our analysis is challenged by the reality that government policy may not automatically receive compliance from the populace. Obvious examples include the emergence of the ‘anti-masker’ movement in the USA and the progression of Black Lives Matter protests despite social distancing restrictions [31]. These highlight the need for public ownership and behavior change, which is arguably more likely if the strategy is appropriate for the country in question [32,33,34,35]. Thus, beyond responding rapidly, governments must also select interventions that are appropriate for their specific social, health, cultural and economic reality [36,37,38].

Amid a second wave of infections, the importance of response timing is critical. The global community now has access to significant data and research, which should (in theory) support the mobilization of more effective responses to the pandemic. Although questions remain and prophylactics and treatments are still being trialed, each country is better equipped to understand which measures have (or have not) been effective. The data here suggest that countries facing a second wave should implement stringent interventions when infection rates begin to rise.

### 4.6. Study Limitations and Further Considerations

The OXCGRT stringency index is a ‘low resolution’ composite measure that does not account for nuances in sub-national government responses, which may be significant where state-level measures vary widely between jurisdictions (e.g., in the USA). While this large-scale data aggregation may mitigate the over- or misrepresentation of certain indicators, it may also fail to capture important data because it cannot account for variations in local contexts or the specific effects on the implementation of high-level policy changes. This work assumes that measures imposed by governments are successfully implemented and result in real action on the ground, which may not be the case in certain regions. Further, it relies on the accurate reporting of both restrictions and infection rates. However, case detection might vary between jurisdictions and depend on various factors, including the type of test used, how the samples were analyzed, whether mass screening was implemented and how often people were tested. We are also unable account for barriers to compliance and/or the activation of a response because these factors are also unique to each country and thus, too diverse for the general analysis performed here, particularly given the composite nature of the stringency index and sub-national variation between jurisdictions within each state. Finally, the sample countries are distributed across both hemispheres and thus, the data analysis included countries experiencing both the height of summer and the depth of winter (June–July). The potential effects of seasonality on viral transmission and human behavior were not examined but may be significant, particularly during summer, when social gatherings, vacations and outdoor activities may influence people to gather in public spaces despite social distancing rules. However, our analysis found no significant relationships between average temperature, infection rates and GDP (Table 2 and Appendix B). The effect of climate on viral transmission is under investigation by others but reports thus far are conflicting [39,40], and hence we would be cautious in interpreting any connections between climate and transmission until further information is available.

## 5. Conclusions

COVID-19 pandemic responses require governments to maintain a delicate balance between physical and fiscal health. Highly restrictive interventions may reduce infection rates, but have an immediate economic impact. Stringency has a greater effect on GDP than on transmission in the short term and this work demonstrates that the timing of the response matters more than how stringent it is. It is now becoming clear that the COVID-19 pandemic is not a short-term phenomenon. Thus, in balancing human and economic health, governments implementing highly stringent restrictions (or not!) must consider the long-term implications of their policies. Trading human for economic health by delaying interventions may benefit a country in the short term, but impose long-term burdens on healthcare systems, global trade and workforce stability. Unfortunately, one size does not fit all, and responses must be suitable for their contexts: stringency is not inversely proportional to infection rates and low-stringency responses may not mitigate economic damage. These data emphasize the importance of timing, particularly since the longer-term consequences of the pandemic are yet to be fully realized. For now, governments that take appropriate action swiftly are more likely to decrease transmission—provided that their responses are both appropriate for the unique reality of the country and supported by its populace.

## Figures and Tables

**Table 1:** Characteristics of Oxford Stringency Index for the focus countries from January to July 2020.

Country	Max. Score	# Days to Respond to First Local Case	# Days to Max. ^a^	# Days at Max.	Response Escalation ^b^ (Days)	AUC ^c^	AUC_av_^d^
Asia							
China	82	49	86	47	81	12,391	66
Japan	47	−8	107	28	100	5705	31
Europe							
Finland	60	−3	88	18	61	6323	38
Norway	80	−27	84	27	53	7150	44
Sweden	46	37	95	70	26	4861	39
UK	76	2	86	48	53	8364	52
America							
USA	73	12	81	86	48	8544	53
Colombia	91	−46	118	9	97	10,168	59
Australasia							
Australia	76	0	93	16	68	8153	49
Statistics ^e^							
Median	76	0	88	28	61	8153	49
Range	46–91	−46–49	81–118	9–86	26–100	4861–12,391	31–66
IQR ^f^	20	20	9	30	28	2221	14
ICC ^g^	<0.001	<0.001	0.561	0.391	0.668 *	<0.001	<0.001

^a^ Number of days since 1 January 2020; ^b^ Response escalation is the time taken to reach max SI after a response was initiated; ^c^ Area under curve (AUC) quantifies the stringency level and duration of each pandemic response; ^d^ AUC_av_ = AUC/duration of response; is a measure of average stringency in a response period; ^e^ Statistics were calculated for data from the focus countries (see Table A1 for China and 37 OECD countries); ^f^ Interquartile range (IQR); ^g^ Intra-cluster correlation coefficient (ICC) measures the extent of correlation within continents (* *p* < 0.05). Data were downloaded from [10].

**Table 2:** Average number of daily cases per million people before, during and after periods at maximum stringency.

	Average Daily Cases Per Million People		
	Before Max SI ^a^	At Max SI ^b^	After Max SI ^a^
USA	2.6	72.9	129.8
Colombia	3.0	6.2	16.9
UK	8.4	61.2	27.5
Sweden	17.4	59.2	89.7
Finland	8.4	22.4	18.2
Norway	15.3	32.6	8.7
China ^c^	1.7	0.1	0.0
Japan	1.9	2.3	0.4
Australia ^c^	5.9	6.0	0.9

Case data were downloaded from the Oxford Martin School [12]; ^a^ Daily case rates averaged over a 30-day period; ^b^ Daily case rates averaged over the duration of each country’s period at maximum SI; ^c^ Case rates are presented for the first period at maximum SI.

**Table 3:** Results of negative binomial regression models on total infections in 38 countries from 1 June to 23 July 2020.

Risk Factor ^a^	Rate Ratio (95% CI)	*p*-Value
GDP (per USD 1000) ^	1.024 (1.000, 1.052)	0.078
Democracy index ^	1.042 (1.004, 1.082) *	0.030
Average temperature (Jan–Mar)	1.079 (0.986, 1.181)	0.099
Average temperature (Jan–Jun)	1.076 (0.955, 1.213)	0.228
Population density ^	1.000 (0.996, 1.004)	0.861
Median age	0.850 (0.766, 0.943) *	0.002
Aged > 65	0.941 (0.807, 1.098)	0.442
Aged > 70	0.944 (0.764, 1.167)	0.596
Variable related to Oxford Stringency Index ^§^:		
Maximum scoreb	0.988 (0.962, 1.015)	0.376
Time to respond (to pandemic) ^	1.028 (1.006, 1.050) *	0.013
Time to respond (to first local case) ^b^	1.011 (0.995, 1.027)	0.194
Time to Maximum score ^c^	1.072 (1.023, 1.124) *	0.004
Response escalation ^b^	0.993 (0.972, 1.015)	0.547
Period at Maximum score ^b^	1.004 (0.982, 1.026)	0.725
AUC ^	0.9997 (0.9993, 1.0000)	0.084
AUC_av_^b^	0.983 (0.939, 1.028)	0.448

Data were downloaded from the Oxford COVID-19 Government Response Tracker [10]. ^ Negative binomial mixed-effect model (with significant continental effect); * *p* < 0.05; ^§^ SI measures up to 31 May 2020; ^a^ Univariate analysis unless otherwise stated; ^b^ Multivariate analysis: adjusted for median age; ^c^ Multivariate analysis: adjusted for GDP and median age.

## Appendix A

**Table A1:** Characteristics of Oxford Stringency Index for China and 37 OECD countries from January to July 2020.

Country	Max. Score	# Days to Respond		# Days to Max. ^a^	# Days of Response	# Days at Max.	Response Escalation ^b^ (Days)	Escalation Speed ^c^ (SI/Day)	AUC ^d^	AUC_av_^e^
		To Pandemic ^a^	To First Local Case							
Asia										
China	82	5	49	86	188	47	81	1	12,391	66
Israel	94	27	−26	99	166	6	72	1.3	10,078	61
Japan	47	7	−8	107	186	28	100	0.5	5705	31
Korea	82	31	11	97	162	12	66	1.2	8397	52
Turkey	78	24	−48	102	169	4	78	1	8797	52
Europe										
Austria	85	55	−2	76	138	29	21	4.1	7658	55
Belgium	81	28	−7	80	165	46	52	1.6	8661	52
Czech Republic	82	24	−38	83	169	10	59	1.4	7509	44
Denmark	72	58	0	78	135	28	20	3.6	8077	60
Estonia	78	72	13	89	121	29	17	4.6	6447	53
Finland	60	27	−3	88	166	18	61	1	6323	38
France	91	23	−2	77	170	55	54	1.7	9832	58
Germany	73	24	−4	82	169	43	58	1.3	7729	46
Greece	84	56	−2	83	137	42	27	3.1	8196	60
Hungary	77	59	−32	88	134	37	29	2.6	7908	59
Iceland	54	23	−26	80	170	45	57	0.9	6105	36
Ireland	91	35	−26	97	158	42	62	1.5	9074	57
Italy	94	23	−8	103	170	22	80	1.2	10,333	61
Latvia	66	31	−8	87	162	46	56	1.2	6930	43
Lithuania	87	57	−2	101	136	4	44	2	8084	59
Luxembourg	80	65	4	77	128	34	12	6.6	6904	54
Netherlands	80	66	7	91	127	41	25	3.2	8065	64
Norway	80	31	−27	84	162	27	53	1.5	7150	44
Poland	83	23	−41	100	170	46	77	1.1	8409	49
Portugal	88	26	−37	100	167	8	74	1.2	9191	55
Slovak Republic	87	27	−40	99	166	6	72	1.2	7850	47
Slovenia	90	64	−1	90	129	21	26	3.5	7031	55
Spain	85	31	−1	90	162	35	59	1.4	8730	54
Sweden	46	69	37	95	124	70	26	1.8	4861	39
Switzerland	73	56	−1	77	137	41	21	3.5	7466	54
UK	76	33	2	86	160	48	53	1.4	8364	52
North America										
Canada	75	22	−4	92	171	3	70	1.1	8243	48
Mexico	82	59	−1	90	134	63	31	2.7	8221	61
USA	73	33	12	81	160	86	48	1.5	8544	53
South America										
Chile	89	74	10	185	119	4	111	0.8	8443	71
Colombia	91	21	−46	118	172	9	97	0.9	10,168	59
Oceania										
Australia	76	25	0	93	168	16	68	1.1	8153	49
New Zealand	96	22	−37	86	171	33	64	1.5	7466	44
Statistics										
Median	81.5	31	−2.5	89.5	162	31	57.5	1.4	8118.5	53.5
Range	46–96	5–74	−48–49	76–185	119–188	3–86	12–111	0.5–6.6	4861–12,391	31–71
IQR ^f^	13	34	33	16	34	34	44	1.5	1291	12
ICC ^g^	<0.001	0.195	<0.001	0.742 *	0.195	0.22	0.551 *	0.108	0.076	0.081

^a^ Number of days since 1 January 2020; ^b^ Response escalation is the time taken to reach max SI after a response was initiated; ^c^ Escalation speed = max score/response escalation; considers the rate at which each country increased its stringency; ^d^ Area under curve (AUC) quantifies the stringency level and duration of each pandemic response; ^e^ AUC_av_ = AUC/duration of response; is a measure of average stringency in a response period; ^f^ Interquartile range (IQR); ^g^ Intra-cluster correlation coefficient (ICC) measures the extent of correlation within continents (* *p* < 0.05). Data were downloaded from [10].

## Appendix B

To explore the association between response stringency and the impact on GDP due to COVID-19, we adopted multilevel mixed-effects linear regression models with the assumption of random continental effect to analyze changes in annual GDP growth rates (2019–2020) that were forecast under a double-hit scenario [13]. Likelihood-ratio tests were used to assess the significance of continental heterogeneity in GDP impact (Table A2).

**Table A2:** Results of linear regression models on change in annual growth rate 2019–2020 due to a double-hit scenario (38 countries).

Risk Factor	Increase in GDP in % (95% CI)	*p*-Value
GDP (per $1000) ^	0.052 (0.000, 0.100) *	0.034
Democracy index ^	0.044 (−0.011, 0.100)	0.119
Average temperature (Jan–Mar) ^	−0.031 (−0.153, 0.091)	0.616
Average temperature (Jan–Jun) ^	−0.066 (−0.251, 0.119)	0.486
Population density ^	0.002 (−0.004, 0.008)	0.461
Median age ^	−0.002 (−0.196, 0.193)	0.986
Aged > 65 ^	−0.033 (−0.237, 0.170)	0.748
Aged > 70 ^	−0.027 (−0.294, 0.239)	0.841
Variable related to Oxford Stringency Index ^§^:		
Maximum score ^	−0.076 (−0.135, −0.016) *	0.012
Time to respond (to pandemic) ^^,a^	0.039 (0.001, 0.076) *	0.044
Time to respond (to first local case) ^	0.037 (0.004, 0.068) *	0.026
Time to maximum score ^^,a^	0.020 (−0.030, 0.069)	0.442
Response escalation ^	−0.032 (−0.067, 0.004)	0.080
Period at maximum score ^^,a^	−0.0007 (−0.039, 0.037)	0.970
AUC ^	−0.0007 (−0.0012, −0.0002) *	0.006
AUC_av_ ^^,a^	−0.028 (−0.113, 0.058)	0.526

Note: ^ linear mixed-effect model (with significant continental effect); * *p* < 0.05; ^§^ SI measures up to 6 July 2020; Univariate analysis unless otherwise stated; ^a^ Multivariate analysis: Adjusted for GDP; Data were downloaded from [10,13].

## References

[1] Habib H. (2020). Has Sweden’s controversial covid-19 strategy been successful?. BMJ.

[2] Chutel L. Taking on Covid-19, South Africa Goes after Cigarettes and Booze, Too. New York Times.

[3] McKibbin W.J., Fernando R. The Global Macroeconomic Impacts of COVID-19: Seven Scenarios. SSRN Electron. J..

[4] Bofinger P., Dullien S., Felbermayr G., Fuest C., Hüther M., Südekum J., Weder di Mauro B., Baldwin R., Weder di Mauro B. (2020). Economic implications of the COVID-19 crisis for Germany and economic policy measures. Mitigating the COVID Economic Crisis: Act Fast and Do Whatever It Takes.

[5] Bonaccorsi G., Pierri F., Cinelli M., Flori A., Galeazzi A., Porcelli F., Schmidt A.L., Valensise C.M., Scala A., Quattrociocchi W. (2020). Economic and social consequences of human mobility restrictions under COVID-19. Proc. Natl. Acad. Sci. USA.

[6] Vindegaard N., Benros M.E. COVID-19 pandemic and mental health consequences: Systematic review of the current evidence. Brain Behav. Immun..

[7] Organisation for Economic Co-operation and Development OECD Employ. Outlook 2020.

[8] Lopes C.A., Okamoto S. Stopping Coronavirus—What Does the Evidence say Are the Best Measures?. The Conversation..

[9] Bremmer I. The Best Global Responses to COVID-19 Pandemic. TIME.

[10] Hale T., Angrist N., Kira B., Petherick A., Phillips T., Webster S. (2020). Variation in government responses to COVID-19. BSG Working Paper Series.

[11] Rasmussen S.A., Jamieson D.J. (2020). Public Health Decision Making during Covid-19—Fulfilling the CDC Pledge to the American People. N. Engl. J. Med..

[12] Roser M., Ritchie H., Ortiz-Ospina E., Hasell J. Coronavirus Pandemic (COVID-19). https://ourworldindata.org/coronavirus.

[13] OECD Real GDP Forecast. https://www.oecd-ilibrary.org/content/data/1f84150b-en.

[14] Franks P.W. Coronavirus: Why the Nordics Are Our Best Bet for Comparing Strategies. The Conversation. https://theconversation.com/coronavirus-why-the-nordics-are-our-best-bet-for-comparing-strategies-135344.

[15] Barber R.M., Fullman N., Sorensen R.J.D., Bollyky T., McKee M., Nolte E., Abajobir A.A., Abate K.H., Abbafati C., Abbas K.M. (2017). Healthcare Access and Quality Index based on mortality from causes amenable to personal health care in 195 countries and territories, 1990-2015: A novel analysis from the Global Burden of Disease Study 2015. Lancet.

[16] Eichenbaum M.S., Rebelo S., Trabandt M. The Macroeconomics of Epidemics. https://www.kellogg.northwestern.edu/faculty/rebelo/htm/epidemics.pdf.

[17] Cornwall W. (2020). Can you put a price on COVID-19 options? Experts weigh lives versus economics. Science.

[18] Kirigia J., Muthuri R., Nkanata L., Muthuri N. (2020). The discounted value of human lives lost due to COVID-19 in France [version 1; peer review: Awaiting peer review]. F1000Research.

[19] Mahase E. (2020). Covid-19: What do we know about “long covid”?. BMJ.

[20] Baldwin R., Tomiura E. (2020). Thinking Ahead about the Trade Impact of COVID-19.

[21] Cassim Z., Handjiski B., Schubert J., Zouaoui Y. (2020). The $10 Trillion Rescue: How Governments Can Deliver Impact.

[22] Paterlini M. (2020). ‘Closing borders is ridiculous’: The epidemiologist behind Sweden’s controversial coronavirus strategy. Nature.

[23] Milne R. Coronavirus: Sweden Starts to Debate Its Public Health Experiment. Financial Times.

[24] BBC News, Coronavirus: Sweden’s Economy Hit Less Hard by Pandemic. BBC News.

[25] Thunstrom L., Newbold S.C., Finnoff D., Ashworth M., Shogren J.F. (2020). The Benefits and Costs of Using Social Distancing to Flatten the Curve for COVID-19. J. Benefit-Cost Anal..

[26] Schwarze R. Opinion: The Known and the Unknown Economic and Social Consequences of Pandemics. https://www.undrr.org/news/opinion-known-and-unknown-economic-and-social-consequences-pandemics.

[27] Chemnick J. If 1 Life Costs $10M, Economists Say Keep the U.S. Closed. E&E News.

[28] Lee J., McKibbin W. (2004). Estimating the Global Economic Costs of SARS.

[29] Kamerlin S.C.L., Kasson P.M. (2020). Managing Coronavirus Disease 2019 Spread with Voluntary Public Health Measures: Sweden as a Case Study for Pandemic Control. Clin. Infect. Dis..

[30] Studdert D.M., Hall M.A. (2020). Disease Control, Civil Liberties, and Mass Testing—Calibrating Restrictions during the Covid-19 Pandemic. N. Engl. J. Med..

[31] Dave D., Friedson A., Matsuzawa K., Sabia J., Safford S. (2020). Black Lives Matter Protests, Social Distancing, and COVID-19.

[32] Morita H., Kato H., Hayashi Y. (2020). International Comparison of Behavior Changes with Social Distancing Policies in Response to COVID-19. SSRN Electron. J..

[33] Van Bavel J.J., Baicker K., Boggio P.S., Capraro V., Cichocka A., Cikara M., Crockett M.J., Crum A.J., Douglas K.M., Druckman J.N. (2020). Using social and behavioural science to support COVID-19 pandemic response. Nat. Hum. Behav..

[34] Rambaree K., Nässén N. (2020). ‘The Swedish Strategy’ to COVID-19 Pandemic:Impact on Vulnerable and Marginalised Communities. Int. J. Community Soc. Dev..

[35] Bargain O., Aminjonov U. Trust and Compliance to Public Health Policies in Times of Covid-19. https://ssrn.com/abstract=3596671.

[36] Muggah R., Florida R. Megacity Slums Are Incubators of Disease—But Coronavirus Response Isn’t Helping the Billion People Who Live in Them. The Conversation. https://theconversation.com/megacity-slums-are-incubators-of-disease-but-coronavirus-response-isnt-helping-the-billion-people-who-live-in-them-138092.

[37] Alon T., Kim M., Lagakos D., Vanvuren M. (2020). How Should Policy Responses to the COVID-19 Pandemic Differ. in the Developing World?.

[38] Friedman S. South Africa Is Failing on COVID-19 because Its Leaders Want to Emulate the First World. The Conversation. https://theconversation.com/south-africa-is-failing-on-covid-19-because-its-leaders-want-to-emulate-the-first-world-142732.

[39] Auler A., Cássaro F., da Silva V., Pires L. (2020). Evidence that high temperatures and intermediate relative humidity might favor the spread of COVID-19 in tropical climate: A case study for the most affected Brazilian cities. Sci. Total Environ..

[40] Sobral M.F.F., Duarte G.B., da Penha Sobral A.I.G., Marinho M.L.M., de Souza Melo A. (2020). Association between climate variables and global transmission oF SARS-CoV-2. Sci. Total Environ..

